# Lectures on the Diseases of Women

**Published:** 1858-07

**Authors:** 


NORTH AMERICAN

### MEDICO-CHIRURGICAL REVIEW.

JULY, 1858.

### Analytical and Critical Reviews.

Art. I.—*Lectures on the Diseases of Women.* By Charles West, M.D., Physician-Accoucheur to St. Bartholomew's Hospital, and Physician to the Hospital for Sick Children. Part I: Diseases of the Uterus. Philadelphia: Blanchard & Lea, 1857.

We confess a decided preference for courses of lectures, as a form of elementary books, upon all the clinical branches of medicine. Something may be sacrificed in the way of methodical detail; it may not allow of that completeness necessary to exhaust a subject: but, on the other hand, the reader seems to be brought into closer relation with the writer, more into the position of a hearer; he is more easily carried step by step into the subject; his attention is better fixed, his mind less easily fatigued, and special points of diagnosis, pathology, or treatment, are more strongly impressed upon his memory.

The author, to whose work we now call attention, is well known as a successful writer in this style; his course of lectures upon the *Diseases of Infancy and Childhood* has long been before the medical public of this country, and has met with that favor to which its merits entitle it. As a writer, Dr. West stands, in our opinion, second only to Watson, the "Macaulay of Medicine;" he possesses that happy faculty of clothing instruction in easy garments; combining pleasure with profit, he leads his pupils, in spite of the ancient proverb, along a royal road to learning.

A work upon diseases of women was to be expected from his pen; indeed, in presenting it he has but fulfilled a duty he owed to the profession. We do not mean to intimate that we receive his work thanklessly, but to say, that, having shown such abilities as a writer on diseases of children, he was in duty bound to continue his labors, and, in practice, the diseases of women stand in close relation to those of children; that if the investment of his talents in the Children's Hospital produced such rich returns, the profession has a right to expect, from his position at St. Bartholomew's, a harvest as much more abundant as the field of cultivation is larger. There is no similarity between the two institutions,—the Children's Hospital of London is but a small establishment, comparing very unfavorably with similar hospitals on the continent, and unworthy of a city which is probably unequalled in the extent of its public charities; but St. Bartholomew's is one of the oldest, largest, and richest hospitals of the British metropolis, an unbounded field in which to pursue any branch of medical research.

The position of the author in relation to the many questions still undecided in the pathology and treatment of female diseases, may claim a moment's attention before proceeding to a consideration of the work itself. To some it may seem surprising that there are so many disputed points and such wide differences of opinion upon a class of diseases some of which are daily under our observation; but such surprise must cease when we reflect how lately the greater part of our knowledge, not only of the diseases, but of the anatomy and physiology of the female sexual organs, has been attained; in the rapid advance of a science some will be left behind, and those who keep up will find themselves occupied in entirely different ground from that on which they stood but a short time previous. There is nothing, then, surprising in these differences: from the doctrine that an organ of such dense structure as the uterus could not be liable to inflammation, it was natural that opinion should pass to the other extreme, of considering it one of the most frequent and most important uterine diseases; from entire ignorance of the influence of the ovaries on menstruation, it was not surprising that all irregularities of this function should be attributed to them as soon as their importance in the economy became known; from the increased knowledge gained by means of the speculum, it was to be expected that some would find no possibility of treating uterine diseases without it. Again, simplicity in medicine is exceedingly tempting; any theory which will easily explain all deviations from health is sure to find believers; any mode of treatment which cures many ailments by a single method will certainly find followers. The department of female diseases had its full share of these theories and these modes of treatment, while time enough has not yet elapsed to prove the truth or value of each. In attaining this end, in separating the good of every novelty from the bad and the indifferent, in placing a true estimate upon new modes of treatment, we look upon the work before us as a valuable aid. He is evidently no partisan; has no case to make out on one side of a question or the other; you feel, as you read, that facts are not made to bend to any preconceived theory of the writer's; that he has no prejudice to satisfy when treating of views and opinions with which he cannot agree. He does not merely give a dogmatical opinion upon questions at issue, but gives facts to support it, and the grounds of his decision in full; he states the arguments of his opponents with most scrupulous fairness, and bears cheerful testimony to any improvements which they may have originated. His work is one which will not satisfy the extreme on either side, but it is one that will please the great majority who are seeking truth, and one that will convince the student that he has committed himself to a candid, safe, and valuable guide.

The first two lectures of the work are introductory, upon the symptoms of diseases of women in general, and the modes of examination necessary in investigating them. The three ways in which these diseases manifest themselves are by "causing disturbance of function, alteration of sensibility, or change of structure," and the consideration of these points occupies most of these two lectures. The modes of examination bring us at once upon a question of dispute—the value of the uterine sound as a means of diagnosis—and afford an instance of some of those characteristics we have already mentioned, the value of this instrument being one of the many points upon which opinions are divided,—some believing it an invaluable addition to our means of diagnosis, others having no belief in its merits but great fear of the dangers attending its use. The latter is the opinion of so eminent a man as Scanzoni. Dr. West says,—

"The information which this instrument places within our reach is often extremely valuable, and of a kind such as otherwise we could not obtain at all, or could arrive at only very slowly, and by frequently repeated examinations. If in a patient suffering from frequent hemorrhages we ascertain the uterine cavity to be greatly increased in size, our immediate conclusion is that the womb contains some foreign body, as a polypus or fibrous tumor, the presence of which has excited, and serves to keep up the bleeding. If we doubt whether a tumor proceeds from the womb or its appendages, or from some other part within the pelvis, the sound enables us to estimate the weight of the organ, and so strengthen the inference drawn from this experiment, by completely isolating the womb from the tumor, and thus ascertaining positively their independence of each other. Or, lastly, if the uterus be bent upon itself either forward or backward, the diagnosis of this condition, which once was a matter of much difficulty, is now arrived at with facility; by introducing the sound with its concavity directed toward the swelling we detect it per vaginam, and observing whether or not this swelling disappears on turning round the instrument. * * * I shall hereafter have to refer on many occasions to this valuable aid to diagnosis. The uterine sound, indeed, is not always applicable, nor does it when used always clear up our doubts; but I do not remember any instance in which a diagnosis, based on the information which it afforded, turned out afterwards to be erroneous."

His remarks are equally judicious in regard to the merits of the speculum, an instrument which has been much abused, in two senses of the word. He neither denounces it nor lauds it, but acknowledges that we owe much of the progress which has been made in uterine pathology to those who were not content with the means of investigating uterine disease previous to its discovery; not all, however, part of it being due to the impulse it gave to the study of the subject.

" * * * The speculum enables us in many instances to decide at once, and with certainty, upon the nature of a case, which otherwise we should have understood only after long and careful watching, to discover some minute polypus which fingers alone would not have detected, to determine the source of a profuse leucorrhœal discharge, and to decide whether it is furnished by the cavity of the womb or the walls of the vagina; or, from the redness, congestion, or abrasion of the os uteri, to infer the state of the womb generally, and thus to conduct our treatment upon the sure ground of positive observation, not upon bare presumptions. At the same time, however, that I hold the speculum to be in many cases of most essential service, I think that the endeavor of all of us should be to ascertain the minimum of frequency with which its employment is necessary. This is to be done not by decrying the instrument, still less by attributing dishonest motives to those who use it, but by soberly and honestly trying to test the value of the information which we derive from it, and learning to discriminate between those appearances which the speculum discloses that are of moment and such as are of no importance."

The three following lectures are upon the disorders of menstruation,—a subject upon which there is little new, as, indeed, but little could be expected. Still, there are some points deserving notice, and all these disorders are considered at length, and in a plain, practical manner, which leaves little to be desired as a guide to practice. Amenorrhœa from malformation of the sexual organs, is stated to be very much more rare than would be presumed from the attention which has been bestowed upon the subject by writers. Absence of the ovaries is rather to be presumed than proved during life; the author has seen only two cases where there was reason to suspect some defect of development of these organs. He has also seen but one case of absence of the uterus, and not one where the menstrual flow was prevented by occlusion of the os or vagina; such cases, he thinks, must be extremely rare. Passing on to the cases demanding the physician's rather than the surgeon's care, he takes pains to impress the fact that postponement of the appearance of the menses does not warrant treatment, and need not occasion anxiety,—a certain amount of variety being observable in regard to this function, as well as to some others, while a serious illness just before the critical age always delays it; he also shows that its establishment is generally *gradual*, presenting in this particular a striking resemblance to dentition, and that the periodically recurring leucorrhœa—the *menstruæ albæ* of old writers—is not abnormal. But after all these cases are deducted there yet remain "a number of instances where the non-accomplishment of the menstrual process, at the time when the changes of puberty are usually completed, is the prominent symptom of disordered health, and seems to be the chief occasion of all the various forms of illness with which it may be associated." These are divided into two classes, according to the symptoms of plethora or anemia which accompany them,—the former, however, having a strong tendency to pass speedily into the latter,—a saving clause, which probably explains why we so rarely meet with it in practice. We cannot follow the author through his very excellent description of these different forms of disease: the connection between the local and constitutional disease, the establishment of vicarious hemorrhage and of chlorosis; nor is there much in the treatment to detain us. He lays down the principle very plainly, that "when no sign of menstrual effort shows itself, then no local measures are indicated;" and shows that he does not believe health alone to be found in the preparations of Apothecaries' Hall.

"In cases where general debility characterizes the patient's condition, tonics, in the widest sense of the term, are indicated; and by them I understand not merely tonic medicines, or preparations of iron, although they will almost always be appropriate, but the tonic influence of pure air, healthful pursuits, and exercise short of fatigue."

In cases where there is a distinct effort at menstruation, after the general health has received due attention, special treatment may be commenced. All harsh and violent remedies are opposed; he follows only those measures which can do no injury, and rejects highly stimulating general and local remedies, such as savin, turpentine, and cantharides, or injections of liq. ammon. in milk, or the application of nitrate of silver by a *porte-caustique.* Benefit has been found from electricity in some instances, but it is an uncertain remedy; and ergot does not seem to have any power except on the muscular structure of the uterus.

In connection with these cases, as well as in the treatment of suppressed menstruation, the influence of habit is a point which the physician should constantly bear in mind; but it is rarely, indeed, that he can have the opportunity of superintending the establishment of the function, and by following a course of treatment month after month during the menstrual period, see that the flow is not only regular in its occurrence, but natural in other respects; unless actual disease of some severity be present, the physician is not consulted, and thus, as our author states, a habit of painful, scanty, or profuse menstruation is fixed for life.

We are very glad to forsake the old division of the subject of menorrhagia for one which we think far more scientific, as it is more simple: viz. according as it depends, "1st, on some cause seated in the constitution generally; 2d, on some affection of the sexual system." To distinguish to which of these classes a given case belongs, is of the highest importance in regard to treatment, as sometimes the disease is but the local sign of a grave constitutional ailment, and sometimes the symptom of organic uterine affection. As an instance of the former we quote the following:—

"Cases are sometimes met with in which an altered state of the circulating fluid, such as even our rough chemistry can detect, coexists with and appears to be the exciting cause of menorrhagia. In cases of granular degeneration of the kidneys, menorrhagia is far from being an uncommon occurrence. The altered, attenuated blood seems to escape more readily than natural from the uterine vessels, when they are congested at the return of a menstrual period; and three or four cases of supposed disease of the womb have come under my notice, in which the most careful examination could detect no local cause for the profuse menstruation, but in which the urine was discovered to be loaded with albumen. The hint which this fact suggests as to the expediency of examining the urine, even though no symptoms should seem to point to the existence of renal disease, is worth remembering, and the test-tube will help to clear up many an obscure case of supposed uterine ailment. You are not to be specialists, even though chance should lead you to have most to do with one special class of ailments, but you are to be physicians, and in proportion as you learn to estimate aright the influence of the disorders of one part upon the functions of another, will you be likely to prove good and successful practitioners in the treatment even of local diseases."

Whatever may have been the origin of the diseases, there are always two indications to be fulfilled: arrest of the present hemorrhage, and removal of the cause. The measures necessary to effect the latter must, of course, vary widely according to the case, and for the former we need only give the opinions we find expressed upon a few special remedies; as emanating from a practical and experienced man they are of value. The general course of treatment necessary having been pursued, there are four astringent remedies to which he resorts,—alum, gallic acid, acetate of lead, and matico. Of these the second and last are but little used in this country, so far as we know; they are not generally procurable, while the lead is a remedy which we believe to be most frequently resorted to and relied upon. His opinion of their relative merits is as follows:—

"Of the four remedies which I have just mentioned, the gallic acid and the matico are those in which I have the greatest confidence, while I place the least reliance on the acetate of lead. I do not know, however, of any special indication by which we can judge beforehand of the probability of one or the other remedy proving especially applicable in any particular case, but am accustomed to employ each in succession, provided one should fail to produce the desired effect."

It may be well to mention that he gives a formula for the exhibition of lead in a liquid form, and in his work on *Diseases of Children* he expresses a preference for this mode of administering the remedy.

In regard to ergot, the author states that he has not been able to discover that it possesses any virtues in controlling uterine hemorrhage, independent of that which it exerts in producing muscular contractions of the organ; he prefers the infusion to all other preparations of the drug. The use of digitalis as a remedy in this disease is alluded to, but he has had no experience with it; he gives the opinion of Dr. Lee, who has employed it extensively, and believes that it is as powerful and certain in controlling hemorrhage from the uterus, as ergot is in exciting the muscular contractions of that organ. We regret that we do not find any mention of Indian hemp (*Cannabis Indica*) in this disease; it is attracting attention, and is highly spoken of by Churchill in the last edition of his *Theory and Practice of Midwifery*, London, 1855.

Under the head of dysmenorrhœa we find described the neuralgic, mechanical, and congestive, with rheumatic and membranous as allied to the latter. Especial pains are taken to impress the fact that progress in anatomical knowledge has entirely done away with the notion that this membrane is a product of inflammation; its production depends only on an exaggeration of the process which normally takes place, and it thus furnishes another instance of the narrow line which separates the domains of pathology from those of physiology. The author is almost as skeptical in regard to dysmenorrhœa depending upon mechanical causes as amenorrhœa; he has seen one case where the canal was narrowed, and in which the suffering commenced after a labor some years before; he believes that generally an undeveloped condition of the organ is the true cause of the narrowed canal. There are some remarks upon this subject too practical and interesting to pass by.

"An impression has, of late years, been gaining ground, that this form of dysmenorrhœa is very common, and mechanical means of treating it have accordingly come very much into vogue, to the neglect, it is to be feared, in many instances, of those internal remedies by which painful menstruation is in general much more appropriately treated. One circumstance, which I believe to have much contributed to the support of this opinion, is the fact that, on introducing the uterine sound, an obstacle is very often encountered at the internal os uteri to the passage of the instrument into the cavity of the womb. That this obstacle is, however, perfectly natural, can be readily ascertained in the dead subject, since even after the removal of the uterus from the body, a bougie which passes with ease along the cervical canal will then encounter a resistance such as can often be overcome only by considerable effort, or perhaps not at all, though a smaller bougie will pass at once with perfect facility, and the uterus, when laid open, will be found to be perfectly healthy. The constriction in this situation, which is found to be so considerable even after death, was, doubtless, in these and many other instances far more considerable during life, and yet in spite of it the history of such persons often gives no account of difficult or painful menstruation. Nor, indeed, need this surprise us, for the discharge takes place, during menstruation, not in a continuous stream as the urine flows from the bladder, but oozes from the interior of the womb, the blood escaping drop by drop from the os uteri. If the aperture be so small as scarcely to allow this to take place, menstruation, no doubt, may be rendered very painful; and just as when stricture of the urethra exists, the bladder and ureters and kidneys become irritated and disturbed in the performance of their functions, so it is quite conceivable that a similar state of the cervix uteri may exert the same influence on the function of that organ, and render the menstrual flux scanty in quantity and morbid in character, as a consequence of the difficulty in its discharge. A slight amount of unbiassed observation, however, will teach you that such a contraction of the os or cervix uteri as to impede the discharge of the menses *guttatim* is very unusual; while it will further show that, in the majority of cases in which this condition really exists, the narrow cervix is only a part of the evil,—that the neck of the womb is small because the organ is altogether very undeveloped."

In the treatment of the neuralgic form of dysmenorrhœa the hot hip-bath (96°–98°) is recommended to be frequently repeated, and for the direct relief of pain to resort to all other remedies before using the preparations of opium, on account of the severe gastric disturbance sometimes following its use; compound spirits of ether with chloric ether, hyoscyamus with camphor, and Indian hemp; two objections, however, are made to the latter, which must militate against its use for any disease,—it is stated to vary much in strength, and the susceptibility of patients to its effects is also said to be very different. The inhalation of chloroform or ether sometimes gives permanent relief, and the application of the former to the hypogastric region is also useful; but nothing is said of the introduction of its vapor into the vagina, from which proceeding Scanzoni says he has seen immediate and perfect relief of the pain, even when only applied for a few minutes.* Nor is there any notice of belladonna as being of service in this distressing disease—a remedy which, in our hands, has acted very efficaciously many times. For the congestive form of the disease the indication is very plain; in these cases, where, as the author expresses it, the congested womb aches until nature bleeds it, our course plainly is to imitate nature, and relieve the oppressed organ by artificial

* Lehrbuch der Krankheiten der Weiblichen Sexualorgane. Wien, 1857.

means. Leeching the uterus itself has proved most efficacious, but it is a proceeding which, for various reasons, cannot be resorted to in this country out of the larger cities, and cupping to the sacrum, or at best scarifications of the neck of the uterus, must be used as substitutes. In those allied cases depending upon the rheumatic diathesis, "colchicum is often of much utility, and, during the paroxysm, twenty or thirty minims of the tincture, with small doses of laudanum and of antimonial wine, will often give more relief than any other remedies, and prove especially useful when large doses of narcotics would be of no service;" but the principal part of the treatment for the cure of these cases must be carried on during the intervals. His opinion having been given upon the rarity of mechanical causes of dysmenorrhœa, but little is necessary upon its treatment; in regard to instruments for dilating or incising the cervical canal, he says he is "perfectly at a loss as to the principle upon which these instruments are recommended. If the cervix uteri be wide enough to admit them, I do not see how its narrowness can offer a mechanical impediment to the escape of the menses." Such instruments are much less frequently used now than a few years ago.

In the sixth, seventh, and eighth lectures, the subjects of hypertrophy and inflammation of the uterus are considered, with their treatment. We shall not enter upon this subject, because the author's Croonian Lectures have been before the profession for some time, and, excepting what relates more immediately to practice, what is given in the work is to be found at greater length in them. Nor do we believe there is any necessity for a repetition of the arguments against the view that chronic inflammation and ulceration of the os and cervix uteri are the sole or chief origins of all female complaints; if the *coup de grâce* has not been given to that doctrine in the lectures above alluded to, we need not expect to see it given now, or, indeed, hereafter.

In connection with the subject of inflammation of the uterus, we will quote a paragraph from one of the lectures on cancer, not only as relating to it, and as one example of the great progress which has been made in uterine pathology, but particularly as an instance of the author's extreme candor and fairness in giving full credit for any improvements, even though they may have been made by those against whose doctrines he has shown himself an active and powerful opponent:—

"To those practitioners and writers, both English and foreign, who have taken the most active part in the study of the inflammatory affections of the neck of the womb, and whose investigations have led them, as some believe, (and I confess myself to be of that number,) to an exaggerated estimate, both of their frequency and of their importance, we yet owe a debt of gratitude for the light which they have thrown on this disease, which outweighs many overstatements and cancels many errors. Cancer of the uterus used, before their time, to be described as a disease slow in progress, continuing in its first quiescent stage of scirrhus not only for months, but for years, and then excited, by one knows not what cause, to activity, passing into the stage of ulcerated carcinoma, and thus, at its close, quickly destroying the patient. It sufficed then for the neck of the womb to be hard and painful, and somewhat enlarged, for the suspicion of malignant disease to be entertained, and for years of causeless anxiety to be entailed upon the patient. Such and such like were the results which followed from confounding the consequences of inflammation and of kindred processes, with the changes which the deposit of the elements of cancer brings about in the affected part."

The subject of flexions of the uterus is of interest, on account of the different views held by eminent men in regard to their pathology,—the question whether the symptoms which accompany them are caused primarily by the disease, or are occasioned by the state of the uterus they induce,—and the extent to which mechanical treatment is beneficial in remedying the evil. Our author states that he follows Virchow's explanation of the manner in which these flexions take place, who believes that the cause is to be found in the anatomical relations of the parts; the neck of the womb being firmly connected with the posterior and lower part of the bladder and the body being movable, any force acting upon the latter part, whether slowly and constantly, or more suddenly, tends to bend the organ upon itself, and produce the disease in question. Now, in turning to Scanzoni's work, (p. 71,) already referred to, we find that he attributes to Virchow the doctrine that ante and retro-flexions are produced by peritoneal adhesions, and he devotes considerable space to combatting a view, which we think disproved by the observation of a single case, where the disease was present without such adhesions, and he refers to several such. Again, Scanzoni himself makes use of the anatomical relations and connections of the cervix uteri compared with the mobility of the body, to explain the fact that these flexions, when they occur in women who have never borne children, are more extreme than in others—the stretching which the parts have undergone by child-bearing having produced a laxity of the structures which allows, in the one case, a certain amount of version to take place with the flexion which cannot occur in the other. Without reference to the original authorities we cannot reconcile the discrepancy, nor decide which is the true authority; it is equally difficult to suppose Scanzoni unacquainted with the true views of his eminent countryman, or that a man so well read as Dr. West should have made the error.

The relative frequency of anterior and posterior versions and flexions of the uterus is also an undecided question. The author's experience of thirty-five cases, in only nine of which was the dislocation anteriorly, does not agree with the statement of Rokitansky, who asserts the former to be the more frequent. The statistics of Valleix and of Mayer show that both forms occur with about equal frequency. Our author believes, however, that anteversion is frequently overlooked.

The study of the causes of these forms of disease introduces some most interesting points, which we can only glance at. As they occur in great disproportion during the child-bearing age of woman, their cause must be sought for in circumstances connected with the highest functions of the sexual organs. Therefore, very early marriage, frequent abortions, or child-bearing, will be found to have preceded the disease generally; whatever tends to check the process of involution of the womb after pregnancy must be a powerful cause of the disease; thus, abortions are more likely to produce it than labor, because the patient resumes her ordinary occupations sooner after the former than the latter, too often deeming a miscarriage a matter of no moment, and thus subjecting herself to chronic inflammation of the uterus, which effectually checks its return to a natural state; efficient pains during labor have also a great effect upon the involution of the uterus, and in cases of flexions of the uterus, a large proportion are found to have been delivered by instruments; but, of all causes, the suckling of the infant is the most efficient in producing perfect uterine involution, and of those suffering from flexions, a large majority have not fulfilled this duty of maternity. We know of no more striking instance than this of the beautiful harmony which reigns through nature,—a harmony which we perceive more clearly the more we understand her operations. In this instance, until deficient involution of the uterus after pregnancy has been recognized as a cause of various forms of disease of the organ, we failed to perceive the importance of other circumstances, the presence or absence of which tended to continue it in its hypertrophied and congested condition, ready at any moment to be attacked by more severe disease.

There are even more discrepancies of opinion in regard to the symptoms of flexions of the uterus, than in regard to other points concerning them,—or, rather, as to how much the symptoms depend primarily upon the mechanical condition of the organ, or are only secondarily connected with it. The question involves another, in regard to the extent to which reposition of the organ, and its retention by mechanical means, will cure the patient of her ailments. The remarks of the author, in this connection, are highly judicious—entirely free from any feeling of a case to be made out on one side or the other. Excessive and painful menstruation, leucorrhœa, pain and difficulty in micturition and defecation, and pain in the pelvic region generally, accompany flexions of the uterus.

"In these symptoms it is obvious that there is much that of itself cannot be regarded as pathognomonic of one uterine affection rather than of another, since they constitute just that train of ailments, which, in varying combinations and with varying intensity, we meet with in almost every disorder of the womb. To this, however, it would not be right to attach much importance, since the uterine ailments that manifest themselves by some one characteristic symptom, or by characteristic combinations of symptoms, are very few indeed. * * * * The symptoms are like the alarm-bell, which gives notice of a something wrong, and serves to awaken attention; it is not fair to expect that they should at once inform us not merely what part suffers, but what the exact cause is on which those sufferings depend."

The author next goes on to caution the student against deciding that these flexions of the uterus are of no importance, merely because they are sometimes proved to be present where not a sign of uterine disease has ever presented itself; and he states that cancer not unfrequently makes considerable progress, and fibrous tumors often attain considerable size, without giving rise to any symptoms. Again, as to the liabililty of overrating their importance:—

"There is a French phrase which expresses excellently well the character of those in whom both these misplacements, and other uterine ailments, are generally attended by the most urgent symptoms: they are persons *qui s'écontent vivre*—who watch themselves live; and the ailments, of which another would be barely conscious, are to them sources of exquisite torture. The ailment may be a real one, and yet it may be the wiser and more hopeful course to try to remedy the state of constitution which exaggerates the patient's sufferings, rather than to meddle with the local affection that excites their present manifestations."

Rejecting those cases where the patient is over-sensitive, as well as those in which the disease exists without giving rise to any symptoms, our author believes that many yet remain whose sufferings, or many of them, may be safely attributed to the uterine flexion. Our readers will have already anticipated that for the relief and cure of these he does *not* resort to mechanical means, and rejects the intra-uterine pessary. But a method of cure introduced and practiced by so celebrated an authority as Dr. Simpson, and sustained by such men as Valleix, of Paris, and Kirvisch, of Prague, is not rejected without an examination of its merits, and a full statement of the arguments against its use. These occupy several pages of the work, and we must refer to it for the details. Dr. West has tried the instrument, and found it not without danger as well as inconveniences, and he now contents himself with a plan of treatment which is less showy and less attractive to the vulgar, but which rarely fails to relieve the patient; "to the best of my power, I take care of the general symptoms, and leave the misplacement to take care of itself." Rest in the horizontal position, leeches to the uterus occasionally, if that organ is tender, careful attention to the state of the bowels, strict precautions during the menstrual periods, the administration of chalybeates, the relief of pain, and the cold hip-bath and douche, are some of the items which make up his plan of treatment. There is a striking coincidence between the opinion of Dr. West and that of Scanzoni, as to the value of the intra-uterine pessary in these cases, and a great similarity between their modes of treatment for the relief of the symptoms accompanying flexions. Both testify to the pain and leucorrhœa produced by the instrument, the inconvenience attending its use, and its worthlessness as a remedy for the disease; and both combat the general symptoms—seeking to subdue existing inflammation, remedy imperfect involution, check the leucorrhœal discharge, quiet pain, increase the tone of the pelvic organs, and set the general system in a healthy state, leaving the flexion to take care of itself. The testimony of Scanzoni on the merits of the stem-pessary is especially valuable, as he followed the celebrated Kirvisch, at the head of the Lying-in Hospital at Prague. Kirvisch modified the instrument of Simpson, and used it extensively in his practice, and was one of the warmest advocates of this mode of treatment in Germany; yet Scanzoni says he has examined many women who were a long time under his predecessor's treatment, and had worn his instrument, who were not relieved of the flexion. As to the value of the uterine sound, as a means of diagnosis of these cases, the estimate of Dr. West is much higher than that of Scanzoni.

Besides the five lectures occupied with the subject of flexions of the uterus, and those already alluded to, the work contains three lectures on other misplacements of the organ, four on uterine tumors and outgrowths, and three on malignant diseases. As the subjects we have considered here are no more interesting in and of themselves, and no more important in practice than those to which we have not called attention, so are they no more ably and judiciously treated; we have taken, almost at random, two or three subjects, and, in giving the views of the author upon them, believe we have enabled our readers to judge of the merits of the whole work. Indeed, we do not think we have noticed the best part of the work; if there is any part of it which surpasses in excellence the rest, it is the lectures on cancerous disease; his descriptions are graphic, his pathological views include the latest discoveries, the statistics of the subject are full and instructive, and his measures of treatment, hopeless though these cases may be, are of great practical value.

We anticipate with pleasure the appearance of the second part of the work, which, if it equals this part, will complete one of our very best volumes upon diseases of females. It is impossible, in these days of book multiplication, to say of any one work that it contains everything upon the subject of which it treats, but we can say of Dr. West's, that no library can be complete without it,—the student will find in its pages all that is necessary for him, and the practitioner much to add to his practical knowledge; and both will find pleasure as well as profit from its perusal.

